# Prospective multicentre analysis of the therapeutic approach and prognostic factors determining overall survival in elderly patients with non-small-cell lung carcinoma treated with curative intent.

**DOI:** 10.1259/bjro.20210058

**Published:** 2022-06-13

**Authors:** Jon Cacicedo, Francisco Casquero, Arturo Navarro, Lorea Martinez-Indart, Olga del Hoyo, Andere Frías, Roberto Ortiz de Zarate, David Büchser, Alfonso Gómez-Iturriaga, Iñigo San Miguel, Fernan Suarez, Adrian Barcena, Jose Luis López-Guerra

**Affiliations:** ^1^ Department of Radiation Oncology, Cruces University Hospital/Biocruces Bizkaia Health Research Institute, Vizcaya, Spain; ^2^ Department of Surgery, Radiology and Physical Medicine of the University of the Basque Country (UPV/EHU), Leioa, Vizcaya, Spain; ^3^ Department of Radiation Oncology, Instituto Catalan de Oncología, Avinguda de la Gran vía de l'Hospitalet, 199-203, 08907 L'Hospitalet de Llobregat, Barcelona, Spain; ^4^ Department of Bioinformatics and Statistics, Cruces University Hospital/Biocruces Health Research Institute, Barakaldo, Spain; ^5^ Department of Radiation Oncology, Hospital Virgen Del Rocío, Av Manuel Siurot, Sevilla, Spain

## Abstract

**Objective:**

To analyse patterns of treatment with curative intent commonly used in elderly patients with locally advanced non-small-cell lung carcinoma (NSCLC) and predictive factors of overall survival in routine clinical practice.

**Methods:**

This multicentre prospective study included consecutive patients aged ≥65 years old diagnosed with NSCLC between February 2014 and January 2018. Inclusion criteria: age ≥65 years, stage IIIA/IIIB NSCLC. Treatment decisions were taken by a multidisciplinary committee. Kaplan-Meier curves and log-rank test were used to identify which clinical/treatment-associated variables, or pre-treatment quality of life (QOL) considering EORTC QLQ-C30 (and LC13 module) were predictive of overall survival.

**Results:**

A total of 139 patients were recruited. Median follow-up was 9.9 months (1.18-57.36 months) with a median survival of 14 months (range 11-17 months). In the group>75-year-old patients, the committee recommended chemotherapy and sequential radiotherapy (55.6%) or radiotherapy alone (22.2%), rather than surgery (3.7%) or concomitant radiochemotherapy (16.5%). However, in 65- to 75-year-old patients, surgery and concomitant radiochemotherapy were recommended in half of cases (p=0.003). Regarding multivariate analysis, the risk of death was higher in patients with pre-existing heart disease (p=0.002), low score for physical functioning (p=0.0001), symptoms of dysphagia (p=0,01), chest pain (p=0.001), and those not undergoing surgical treatment (p=0.024).

**Conclusions:**

Patients >75 years received more conservative treatments. Surgery improved survival and should be carefully considered, regardless of patient age. Comorbidities and poor baseline QOL are predictive of shorter survival.

**Advances in knowledge:**

Measuring these parameters before treatment may help us to define a population of frail patients with a poorer prognosis to facilitate decision making in clinical practice.

## Introduction

More than two-thirds of lung cancer cases currently diagnosed are in people over 65 years of age. Indeed, the mean age at diagnosis is 71 years old,^1^ most patients being frail patients with comorbidities that may limit their prognosis and tolerance of treatment.^2–4^ Therefore, it is becoming increasingly important to establish which management approach is most effective in elderly patients with locally advanced lung cancer. A meta-analysis^5^ demonstrated the superiority of concomitant chemotherapy and radiotherapy over sequential chemotherapy and radiotherapy in patients with unresectable stage III lung cancer, with 2- and 5 year survival rates of 36% and 15%, respectively, with concomitant treatment and 30% and 11% with sequential treatment. Nonetheless, there are other treatment options for patients with a poorer general condition, including sequential chemotherapy and radiotherapy^6^ or radiation therapy alone.^7–9^

Several studies indicated that the proportion of patients who receive active treatment for lung cancer decreases with advancing age.^10,11^ Furthermore, in clinical trials, the evidence for the use of different treatment regimens is generally gathered from fit younger patients. Notably, in the meta-analysis of Auperin et al,^5^ most patients included had a good performance status (0–1) and <20% were aged ≥70 years. It is therefore difficult to extrapolate the findings to all patients with non-small-cell lung cancer (NSCLC) who are elderly and have comorbidities.^12–14^ Although there have been small studies in elderly patients,^5,15^ there is no solid evidence regarding tolerance or the importance of patient clinical characteristics to guide us in deciding which is the best treatment option in this population.

Due to ageing is a vague concept, several tools have been designed to predict toxicity and identify which patients would be good candidates to undergo radical treatment or adapted therapy^16^

In this study, we sought to assess patterns of commonly used treatment modalities with curative intent in elderly patients with locally advanced NSCLC and clinical factors predictive of overall survival in the context of daily clinical practice. By combining these clinical findings, we would be able to identify the best treatment for each patient.

## Methods and materials

### Patient population

This multicentre prospective observational study included all consecutive patients aged≥65 years old diagnosed with NSCLC between February 2014 and January 2018. The study was approved by the Ethics Committees of the participating hospitals (xx) and was conducted in accordance with the principles of the Declaration of Helsinki. All patients who participated in the study gave written informed consent prior to inclusion.

*Inclusion criteria:* age≥65 years, a histological diagnosis of NSCLC, locally advanced disease (stages IIIA or IIIB according to the seventh edition of the American Joint Committee on Cancer Staging TNM classification),^17^ receiving radiotherapy with radical intent, with a total prescribed dose of ≥50 Gy (undergoing previous surgery) or ≥60 Gy without a history of surgery, and with or without chemotherapy (concomitant/sequential).

*Exclusion criteria*: previous radiotherapy, recurrence, or previous history of cancer.

### Assessment, treatment and follow-up of patients

Patients were assessed at their first visit through obtaining a clinical history and performing a physical examination. All treatment decisions for these patients were taken by a multidisciplinary committee in each of the participating centres. The treatment options planned were classified as follows: surgery and postoperative radiotherapy (sequentially after postoperative chemotherapy), concomitant radiochemotherapy, sequential chemotherapy and radiotherapy, or radiation therapy alone. Data were collected on the following patient characteristics, categorised as indicated (in parentheses): age (65–75 years vs >75 years old); the Karnofsky Performance Scale (KPS) (<70 vs≥70); smoking habit (into three categories,^18^ smoker, ex-smoker and non-smoker; and also into three categories by smoking history^19^ :≤30 pack-years *vs* 31–75 pack-years, vs >75 pack-years); baseline haemoglobin levels (^20^ (<12 vs≥12 g dl^−1^); pretreatment weight loss (yes *vs* no); alcohol abuse (yes *vs* no); chronic obstructive pulmonary disease (yes *vs* no)^21^ ; pretreatment thromboembolic event (yes *vs* no), heart disease (yes *vs* no), diabetes mellitus (yes *vs* no), type of treatment received (surgery *vs* concomitant treatment *vs* sequential treatment *vs* radiotherapy alone), stage (IIIa *vs* IIIb), radiation dose (≤60 Gy vs >60 Gy), radiotherapy technique (3D conformal radiotherapy *vs* volumetric-modulated arc therapy [VMAT]/intensity modulated radiotherapy [IMRT]).

As it has been suggested that pretreatment quality of life (QOL) has prognostic value,^22,23^ in this study, QOL questionnaire (consisting of the EORTC QOL-C30 and lung cancer module QLQ-LC13) was administered to all patients at baseline. The aim was to assess the effect on survival of patients’ subjective assessment of their own baseline status before treatment.^24^ The EORTC QOL-C30 evaluates QOL in relation to physical, emotional and social factors, considering general level of functioning in oncology patients. The questionnaire is divided into five functional scales (physical functioning, activities of daily living, emotional functioning, cognitive functioning and social functioning), three symptom scales (fatigue, pain, and nausea and vomiting), one global health status domain, and finally six independent items (dyspnoea, insomnia, anorexia, constipation, diarrhoea and economic impact).^25^

The QLQ-LC-13 includes^26^ measures of the symptoms associated with lung cancer (cough, haemoptysis, dyspnoea and pain) and the adverse effects of conventional chemotherapy and radiotherapy (hair loss, neuropathy, sore mouth and dysphagia).

High scores in the symptom scales indicate the presence of symptoms associated with cancer that negatively affect the quality of life. On the other hand, High scores on the global health and functional status scales indicate a better QOL.

For this study, we categorised each of the functional and symptom scores from the questionnaires (EORTC QOL-c30 and module LC13) by the pretreatment score (0–100) into the following categories^27^ :≤33.3 vs 33.3-66.6 vs>66.6 points.

### Treatment

Regarding radiotherapy, immobilisation and treatment planning were performed with patients in the supine position. A vacuum-locked cradle was used for patient immobilisation when deemed necessary. In all patients, a contrast computed tomography (CT) scan was performed with a 0.5 cm thickness, from the atlas bone (C1) to the second lumbar vertebra, approximately, to include the entire neck and the lungs.

Radiation was administered with 3D conformal radiotherapy or VMAT/IMRT using radiological imaging to delineate the gross target volume of the primary tumour (GTV-P) and/or macroscopic lymph node involvement (GTV-N). Any regions of the tumour visible by endoscopy but not seen in the CT images were also included in the GTV-P. The GTV was extended by 6 to 8 mm around the primary tumour and selected lymph nodes to obtain the clinical target volume (CTV), which was, in turn, extended 10 mm laterally and vertically to obtain the planning target volume (PTV). The radical radiotherapy was conventionally fractionated and, in some cases, was preceded by induction or concomitant chemotherapy (doublet therapy with cisplatin or carboplatin) at the discretion of the medical oncologist. Surgery was considered in patients with operable tumours. Thereafter, postoperative radiotherapy was performed in patients found after surgery to have pN2 disease, sequentially after chemotherapy.^28–30^

In designing the treatment, the aim was to use to the minimum dose possible in neighbouring organs at risk: healthy lung tissue, heart, oesophagus, and spinal cord, following the QUANTEC guidelines.^31^

### Follow-up

After treatment, check-ups were performed first at 1 month after the radiotherapy and then every 3 months (including a CT scan of the neck and chest every 3–6 months) by each of the specialists who participated in their treatment (thoracic surgeons, medical and radiation oncologists). Any acute (up to 3 months after treatment) and chronic (from then until after the end of the radiotherapy) toxicity was recorded, using the Common Terminology Criteria for Adverse Events ( *vs* 4.0.).

### Statistical analysis

Continuous variables were expressed as medians and range and categorical variables as frequencies and percentages. To compare categorical variables Chi-square test was used or Fisher exact test when expected frequency less than five.

The primary outcome was the overall survival of the population, analysed using Kaplan-Meier curves. To calculate survival, the time interval considered was from the end of radiotherapy to the date of death (all-cause) or the last follow-up.

Analysis was performed to assess the influence of *clinical characteristics* (age, sex, TNM stage, KPS score, history of heart disease and diabetes, pretreatment weight loss, diagnosed chronic obstructive pulmonary disease, baseline haemoglobin levels, smoking and drinking habits, history of thromboembolism, pretreatment QOL considering EORTC QLQ-C30 and LC-13 scores) and *treatment* (modality, radiotherapy technique, and radiation dose) on patient survival.

Kaplan-Meier curves and log-rank test was used to identify which clinical or treatment-associated variables were predictive of overall patient survival. Subsequently, variables with a *p* < 0.2 in the univariate analysis were included in the Cox multivariate regression analysis (using a non-automatic stepwise procedure), to assess whether they were statistically significant independent predictors (*p* value < 0.05). Cox proportional hazards analysis was performed to calculate hazard ratios (HRs) and confidence intervals (CIs).

The analysis was performed using the IBM SPSS (version 23.0).

## Results

We recruited a total of 139 consecutive patients between February 2014 and January 2017, with a median age of 71 years old (65-88), of whom 123 (88.5%) were males and 16 (11.5%) women. Clinical and treatment characteristics of the population included in the study are described in Table 1. In addition, we described characteristics of our study population according to the age (≤75 vs>75 years). See Supplementary Table 6.

**Table 1.** Clinical and treatment characteristics of the population included in the study

Characteristics	Patients, *n* = 139
	
Age (median and range)	71 years old (65-88)
Karnofsky Performance Scale score ≥70 <70	135 (97.1%) 4 (2.9%)
Sex: Male/Female, n (%)	123 (88.5%)/16 (11.5%)
Histological diagnosis, n (%)	
Adenocarcinoma	44 (31.7%)
Giant cell carcinoma	5 (3.6%)
Epidermoid/squamous cell carcinoma	90 (64.7%)
Comorbidities, n (%)	
Chronic obstructive pulmonary disease	Yes 70 (50.4%)
Diabetes mellitus	Yes 46 (33.1%)
History of heart disease Arrhythmia Hypertensive heart disease Heart failure Ischaemic heart disease Others	50 (36%) 13 (9.4%) 3 (2.2%) 2 (1.4%) 16 (11.5%) 16 (11.5%)
History of thromboembolic event (yes), n (%)	16 (11.5%)
Smoking habits, n (%)	
Smoker	52 (37.4%)
Ex-smoker	80 (57.6%)
Non-smoker	7 (5%)
Pack/years	67 (0–162)
Alcohol abuse, n (%)	
No	86 (61.9%)
Yes	53 (38.1%)
Weight loss, n (%)	
No	91 (65.5%)
Yes	48 (34.5%)
a) Baseline haemoglobin	11.6 gr/dl (range: 6.8–16.4)
Stage, n (%)	
III A	72 (51.8%)
III B	67 (48.2%)
Previous surgery, n (%)	
No	115 (82.7%)
Yes	24 (17.3%)
Radiotherapy technique, n (%)	
3D	117 (84.2%)
Intensity-modulated radiation therapy	1 (0.7%)
Volumetric modulated arc therapy	21 (15.1%)
Radiotherapy dose received (median; Gy)	66 Gy (50–66 Gy)

a Data on baseline haemoglobin was not available for four patients

Based on treatment modality, we classified all 139 patients into one of four groups: 24 patients received surgery and postoperative radiotherapy (17.3%), 38 concomitant radiochemotherapy (27.3%), 67 sequential chemotherapy and radiotherapy (48.2%), and 10 radiotherapy alone (7.2%). We then broke these treatment groups down as a function of age (65–75 vs >75 years old; Table 2): in the >75-year-old patients, the multidisciplinary committee mostly recommended chemotherapy and sequential radiotherapy (*n* = 15, 55.6%) or radiotherapy alone (*n* = 6, 22.2%), rather than surgery (*n* = 1, 3.7%) or concomitant radiochemotherapy and (*n* = 5, 16.5%). In contrast, in 65- to 75-year-old patients, surgery and concomitant radiochemotherapy and were recommended by the committee in approximately half of cases. The differences between these groups were significant (*p* = 0.003) (Table 2).

**Table 2.** Distribution of treatment modality by age

Treatment provided	Age	Age	Total
	65–75 years	>75 years	
Surgery+postoperative radiotherapy (chemotherapy)	23 (20.5%)	1 (3.7%)	24 (17.3%)
Concomitant radiotherapy and chemotherapy	33 (29.5%)	5 (18.5%)	38 (27.3%)
Sequential radiotherapy and chemotherapy	52 (46.4%)	15 (55.6%)	67 (48.2%)
Radiotherapy alone	4 (3.6%)	6 (22.2%)	10 (7.2%)
**Total**	**112** (**100%**)	**27** (**100%**)	**139** (**100%**)

It should be noted that the multidisciplinary committee recommended surgical treatment based on multiple clinical parameters such as performance status, clinical staging, and the presence of comorbidities such as history of heart disease or pulmonary function. Patients undergoing surgery had less chronic obstructive pulmonary disease (*p* = 0.04) and lower T stage (*p* = 0.01).

In addition, (although not statistically significant) patients with previous history of heart disease underwent surgery less frequently (29.2% vs  70.8%).These data are fully described in Supplementary Table 7.

**Figure 1.**
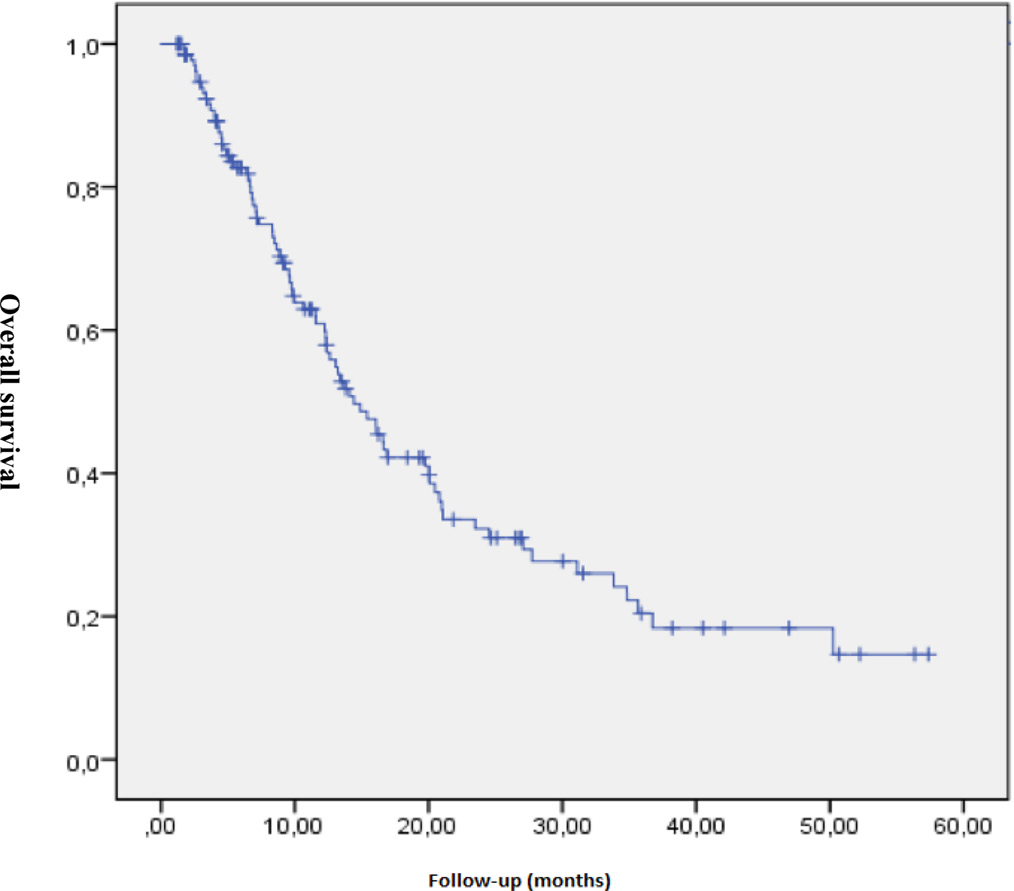
Overall Survival

The median radiation dose was 66 Gy (50-66). The median follow-up was 9.9 months (1.18–57.36 months). The median survival was 14 months (range 11–17 months), and the overall survival rates at 6, 12 and 24 months were 82.7%, 60.9 and 32.3%, respectively (Figure 1).

**Figure 2.**
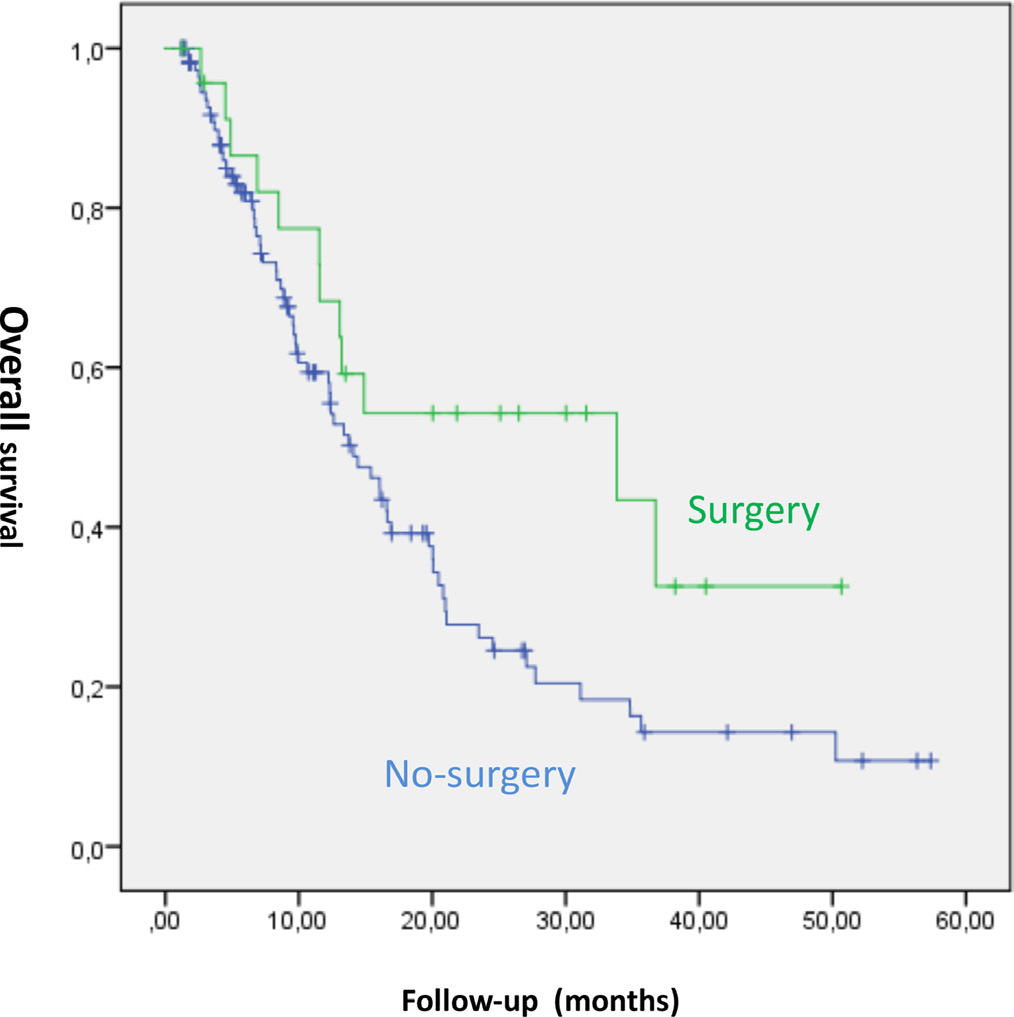
Overall survival regarding treatment modality

Analysing factors with a potential influence on overall survival, the following variables were found to be significant in the Kaplan Meier analysis (Table 3): pack-year history (*p* = 0.049); heart disease (*p* = 0.0001); thromboembolic events (*p* = 0.012); physical, role, cognitive and social functioning (*p* = 0.0001, *p* = 0.0001, *p* = 0.0001 and *p* = 0.003 respectively); fatigue (*p* = 0.017); pain (*p* = 0.029); loss of appetite (*p* = 0.001); dyspnoea (*p* = 0.001); dysphagia (*p* = 0.003); pain in chest (*p* = 0.0001); and previous surgery (*p* = 0.044) (Figure 2).

**Table 3.** Univariate analysis

			95% confidence interval		
Variables	n*	Median survival	Lower limit	Upper limit	*P* value
Patient age					
65–75 years	112	14.09	11.09	17.09	0.275
>75 years	27	14.42	8.42	20.42	
Sex Female Male		12.2 14.4	0 11.6	26.3 27.2	0.989
Karnofsky Performance Scale score					
<70	4	4.27	0	21.27	0.396
≥70	135	14.42	11.28	17.56	
Smoking habits					
Smoker	52	20.04	12.75	27.32	0.270
Ex-smoker	80	13.20	11.18	15.23	
Non-smoker	7	33.84	0	0	
Pack-years					
≤30	16	33.84	2.89	64.78	**0.049**
31–75	62	13.76	11.54	15.98	
>75	61	16.92	8.59	25.24	
Haemoglobin (g/dl)					
<12	78	14.09	11.56	16.62	0.947
≥12	57	16.06	10.91	21.21	
Weight loss					
No	91	12.61	8.15	17.07	0.178
Yes	48	20.46	12.56	28.37	
Alcohol abuse					
No	86	13.76	10.17	17.35	0.492
Yes	53	14.42	10.36	18.48	
Chronic obstructive pulmonary disease					
No	69	16.92	9.59	24.25	0.050
Yes	70	13.07	10.37	15.77	
Diabetes Mellitus					
No	93	13.20	10.69	15.71	0.928
Yes	46	16.06	14.31	17.81	
Heart disease					
No	89	23.49	13.02	33.95	**0.0001**
Yes	50	9.98	6.42	13.55	
Thromboembolic event					
No	123	16.62	11.03	22.21	**0.012**
Yes	16	12.35	7.27	17.43	
Treatment modality					
Surgery (yes)	24	33.84	0.69	66.99	0.07
Concomitant chemotherapy	38	14.09	7.79	20.39	
Sequential chemotherapy	67	12.61	8.52	16.70	
Radiotherapy alone	10	13.37	4.92	21.81	
Physical functioning, C30					
≤33.3	5	4.27	0.81	7.72	**0.0001**
33.3–66.6	23	9.56	1.76	17.35	
>66.6	99	20.07	15.05	25.09	
Fatigue, C30					
≤33.3	56	21.06	14.22	27.89	**0.017**
33.3–66.6	48	12.61	11.35	13.88	
>66.6	25	8.90	0	19.70	
					
Nausea and vomiting, C30					
≤33.3	120	14.88	11.62	18.14	0.111
33.3–66.6	6	14.09	1.28	26.90	
>66.6	3	5.48	0	11.53	
Pain, C30					
≤33.3	91	16.92	10.70	23.13	**0.029**
33.3–66.6	22	9.26	4.45	14.07	
>66.6	13	9.56	2.14	16.97	
Dyspnoea, C30					
≤33.3	69	14.42	10.61	18.23	0.407
33.3–66.6	42	15.40	6.44	24.37	
>66.6	18	9.56	0	22.01	
Loss of appetite, C30					
≤33.3	78	20.99	13.38	28.60	**0.001**
33.3–66.6	27	9.26	1.18	17.34	
>66.6	25	10.64	6.31	14.97	
					
Constipation, C30					
≤33.3	69	20.46	15.29	25.64	0.055
33.3–66.6	39	13.20	10.74	15.67	
>66.6	22	10.64	4.82	16.46	
Diarrhoea, C30					
≤33.3	110	13.37	10.28	16.46	0.450
33.3–66.6	17	34.82	13.38	56.26	
>66.6	3	27.72	0	0	
Financial impact, C30					
≤33.3	98	16.06	10.40	21.72	0.143
33.3–66.6	22	12.22	4.25	20.18	
>66.6	10	0	0	0	
Dyspnoea, LC-13					
≤33.3	104	16.92	11.15	22.68	**0.001**
33.3–66.6	18	8.90	5.41	12.39	
>66.6	4	4.56	0	13.66	
Cough, LC-13					
≤33.3	31	13.37	3.81	22.93	0.300
33.3–66.6	67	16.06	12.10	20.02	
>66.6	30	12.22	4.53	19.91	
Haemoptysis, LC-13					
≤33.3	108	15.40	8.25	22.55	0.103
33.3–66.6	16	13.37	6.16	20.57	
>66.6	5	12.32	0	29.53	
Sore mouth, LC-13					
≤33.3	112	14.42	10.92	17.92	0.689
33.3–66.6	14	9.75	0	0	
>66.6	3	16.06	0	39.04	
Dysphagia, LC-13					
≤33.3	106	16.06	10.28	21.84	**0.003**
33.3–66.6	17	12.38	6.49	18.28	
>66.6	6	3.02	0	8.76	
Peripheral neuropathy, LC-13					
≤33.3	80	13.37	10.07	16.66	0.601
33.3–66.6	31	14.42	9.22	19.61	
>66.6	17	27.07	2.31	51.82	
Hair loss, LC-13					
≤33.3	83	13.07	9.80	16.34	0.084
33.3–66.6	19	13.20	10.06	16.34	
>66.6	26	34.82	0	70.93	
Pain in chest, LC-13					
≤33.3	90	16.06	9.77	22.36	**0.0001**
33.3–66.6	27	16.92	9.40	24.43	
>66.6	12	4.30	2.03	6.57	
Pain in arm or shoulder, LC-13					
≤33.3	94	14.42	7.29	21.54	0.447
33.3–66.6	25	14.88	8.66	21.10	
>66.6	9	10.64	7.47	13.81	
Pain in other parts, LC-13					
≤33.3	54	14.09	11.28	16.90	0.390
33.3–66.6	20	24.54	15.58	33.50	
>66.6	16	13.37	0	28.33	
Total dose (Gy)					
≤60	36	14.88	12.02	17.74	0.720
>60	103	14.42	9.91	18.93	
Previous surgery					
No	115	14.09	10.85	17.33	**0.044**
Yes	24	33.84	0.69	66.99	
Technique					
Others	22	7.32	3.01	11.64	0.166
3D	117	14.88	11.77	17.99	
Stage					
IIIa	67	12.32	8.77	15.87	0.083
Others	72	16.92	10.80	23.03	

No differences in overall survival by treatment modality reached significance (*p* = 0.073), although there was a clinical trend towards higher survival in patients who underwent surgery (see Figure 1). Given this, to assess the role of surgery in the study population, the treatment modalities were grouped into two categories (surgery *vs* other treatment modalities) for the multivariate analysis (Table 3).

According to the multivariate analysis, the risk of death was higher in patients with pre-existing heart disease, a low score for physical functioning, or symptoms of dysphagia and/or chest pain, as well as those who did not receive surgical treatment. These variables are considered significant independent predictors. The results of multivariate analysis are shown in full in Table 4.

**Table 4.** Multivariate analysis

Variable	*p*-value	HR	95,0% CI	
			Inferior	Superior
Cardiopathy (present)	**,002**	2,206	1,334	3,647
Physical Functioning (PF) (>66.66)	**,000**			
PF (33.3–66.6)	**,000**	1.546	2,901	25,155
PF (<33.3)	,170	8.546	,829	2,881
Dysphagia (ref<33.3)	**,038**			
Dysphagia (33.3–66.6)	,571	1,213	,622	2,365
Dysphagia (>66.66)	**,010**	4,276	1,406	13,006
Pain in chest (ref<33.3)	**,001**			
Pain in chest (33.3–66.6)	,657	,863	,450	1,656
Pain in chest (>66.66)	**,001**	4,332	1,883	9,965
Surgery (patients not undergoing surgery)	**,024**	2,236	1,111	4,501

Data on acute and chronic toxicity are summarised in Table 5. We performed subanalysis to assess whether acute and/or chronic toxicity (oesophagitis, pneumonitis, heart toxicity) experienced by the patients was influenced by age (65–75 vs >75 years), dose (≤60 vs>60 Gy), treatment modality, or baseline KPS (<70 vs≥70). By treatment modality, Grade three acute oesophageal toxicity was observed in 2 patients out of 16 treated with surgery and postoperative radiotherapy (after adjuvant chemotherapy), 5/16 treated with concomitant radiochemotherapy, and 1/16 treated with sequential radiotherapy and chemotherapy, while there were no cases in the group given radiotherapy alone, the rate of Grade three oesophagitis being significantly higher among the patients treated with concomitant chemotherapy (*p* = 0.022).

**Table 5.** Acute and Chronic toxicity

Toxicity	Patients, *n* = 139
Acute esophagitis n (%)	
Yes	69 (49.6)
No	70 (50.4)
Acute esophagitis; grade, n (%)	
Grade I	13 (9.4)
Grade II	48 (34.5)
Grade III	8 (5.8)
	
Chronic esophagitis* n (%)	
Yes	18 (12.9)
No	118 (84.9)
Chronic esophagitis; grade, n (%)	
Grade I	4 (2.9)
Grade II	12 (8.6)
Grade III	1 (0.7)
Grade IV	0
Grade V	1 (0.7)
	
Acute pneumonitis; n (%)	
Yes	62 (44.6)
No	77 (55.4)
Acute pneumonitis; grade, n (%)	
Grade I	12 (8.6)
Grade II	33 (23.7)
Grade III	17 (12.2)
	
Chronic pneumonitis; n (%)	
Yes	62 (44.6)
No	75 (54)
Chronic pneumonitis; grade, n (%)	
Grade I	10 (7.2)
Grade II	27 (19.4)
Grade III	21 (15.1)
Grade IV	0
Grade V	4 (2.9)
	
Chronic cardiac toxicity	
Yes	3 (2.2)
No	136 (97.8)
Type of cardiopathy due to chronic toxicity; n (%)	
Heart failure	1 (0.7)
Ischaemic heart disease	2 (1.4)

In addition, we have analysed the influence of treatment technique (3D conventional RT *vs* IMRT/VMAT) in toxicity (including oesophagitis, pneumonitis, heart toxicity). We did not find statistically significant differences. Full data are described in Supplementary Table 8. We did not find any other significant associations between acute or chronic oesophageal, lung or heart toxicity and the aforementioned variables.

## Discussion

The aim of this study was to assess survival and the patterns of treatment among unselected elderly patients with locally advanced NSCLC. The median survival was 14 months (11–17 months), while the 1- and 2 year overall survival rates were 60.9 and 32.3% respectively. These data are similar to those of other studies that have analysed the survival in elderly patients,^32,33^ and even to those found in younger patients with locally advanced NSCLC.^34^

In our study, 27.3% (38/139) of patients received concomitant radiochemotherapy compared to 48.2% (67/139) who received sequential radiotherapy and chemotherapy (48.2%), in line with other specific studies in elderly patients.^12,32,35^

Regarding the treatment modality used, as was expected, the least common approaches were surgery and concomitant radiochemotherapy, especially among the oldest patients (>75 years old). In this latter group, the most common treatment modalities were sequential radiotherapy and chemotherapy or radiotherapy alone. Such a trend towards more interventional approaches involving surgery and concomitant radiotherapy and chemotherapy in patients aged 65 to 75 years, while more conservative treatments (sequential radiotherapy and chemotherapy or radiotherapy alone) are indicated in elderly patients (>75 years old), has been described previously by other authors.^33^

In our study, we have not observed significant differences in survival as a function whether patients received concomitant or sequential chemotherapy, in line with the recent study by Driessen et al..^32^ The survival rate was even similar in patients who received radiotherapy alone and those who received sequential chemotherapy. This finding contrasts with the results of a meta-analysis^36^ which indicated higher survival rates in patients given concomitant radiotherapy and chemotherapy than those given sequential radiotherapy and chemotherapy. Although this might be due to the lack of statistical power in our study, it could also be explained by the trials studied only having included young and fit elderly patients, who are not representative of the patients treated in daily clinical practice.^5,36^

On the other hand, according to our results, it seems important that, regardless of patient age, a clinical committee carefully selects candidates for surgery,^33^ as our multivariate analysis indicates that surgical treatment may influence survival.

Performance status is a well known factor influencing survival in patients with lung cancer. Indeed, in our sample, patients with KPS <70 had a notable tendency towards a shorter survival (median 4.2 *vs* 14.4 months) than those with KPS≥70, although these differences were not statistically significant (Table 3). In our opinion this is probably due to the low number of patients (4 out of 139) included in the study with KPS<70. Moreover, considering that only patients undergoing treatment with radical intent were included in this study, it is reasonable in our consideration, including a majority of patients presenting good performance status.

On the other hand, according to our results, general clinical condition as assessed by measuring baseline QOL (in particular, physical functioning, dysphagia and chest pain) was a significant predictor of survival. This is consistent with the results of various other studies.^23,37,38^ We believe that this is important since QOL parameters can easily be assessed in daily clinical practice before treatment using the EORTC questionnaires (QLQc-30 and LC-13) to facilitate decision making and inform patients about their prognosis. We believe that this is particularly important since our study has produced similar results to those of previous studies in lung cancer,^23,37,38^ but with a focus on a specific sample of patients (elderly patients with locally advanced NSCLC) who are often clinically frail and regarding whom decision making may be a challenge. This finding supports the view that QOL data should be collected in daily clinical practice.

Additionally, it was found that a history of specific comorbidities such as heart disease, which is relatively common in this type of patients, was a significant independent predictor of survival. Grose et al^39^ also noted the importance of comorbidities as an independent prognostic factor in early and advanced stages of lung carcinoma. Therefore, the level of comorbidity should be taken into account to stratify patients and interpret the results of clinical trials, especially in elderly patients.^40^

Regarding toxicity, a direct relationship was not found with age (65–75 vs >75 years old), but was found with the treatment modality, especially in patients who received concomitant chemotherapy, having this group a higher rate of acute oesophagitis. Therefore, when making a treatment recommendation in these patients, we should consider the risk-benefit ratio,^41^ preferences of the patient regarding survival and treatment tolerance, given that in this unselected population of elderly patients the administration of concomitant chemotherapy did not significantly improve survival.^32^

Regarding smoking, our univariate analysis revealed an association between a higher level of smoking (packs/year) and shorter survival, as was reported by other authors.^19^ Nonetheless, this result was not statistically significant in the multivariate analysis. We did not find associations between survival and anaemia, or other clinical parameters related to the treatment such as the technique or radiation dose (Table 3).

Notably, we did not find differences in survival between the oldest patients in the cohort (>75 years old) and those aged 65 to 75 years, age by itself not being found to be determinant in patient survival.

While age itself did not prove prognostic on the multivariate analyses, surgery instead, was a significant factor. This result should be interpreted with caution since only one patient >75 years underwent surgery. Indeed, in the group >75 years of age, only 16 patients could be truly evaluated for surgery (without considering cases with stage IIIB where surgery is clearly not indicated), regarding PS and comorbidities (see Supplementary Table 6). It is, therefore, a small number of patients that could have influenced our results. Future research should focus on predictive patient characteristics to distinguish patients within the heterogeneous older population who can benefit from curative-intent treatment.

After an analysis of overall survival, in line with other studies in younger patients, we affirm that there is no reason to rule out combined treatment for patients based on their age alone.^34,36,42^

However, we should recognise that this study has some limitations. It should be noted that this study was not designed to explore the benefit of a specific treatment approach as it not a randomised trial.

Indeed, the clinical decision may be difficult in elderly patients with lung cancer (usually fragile population) in the absence of high quality data. Considering the risks of surgery and toxicity of chemo-radiotherapy are often increased in the elderly compared with younger patients, patients in this study were therefore, closely scrutinized. Our management recommendations were generally similar to those of general guidelines for the NSCLC population. Careful evaluation was performed to ensure that treatment was guided by patient characteristics, stage, and not by age. All the treatment decisions were based on patient performance status, tumour resectability including T and N stage (see Supplementary Table 7), pulmonary function and presence of comorbidities. The best radical treatment approach was indicated for each patient in a multidisciplinary board. Whenever possible surgery was indicated (±postop-radiotherapy, regarding TNM stage, resectability and comorbidities), followed by concomitant radiochemotherapy or radiotherapy alone.

We also acknowledge that we did not perform a comprehensive geriatric assessment, that might be necessary to provide the best suitable treatment for each patient, and therefore is increasingly been incorporated in oncologic care demonstrating that it can alter treatment decisions.^16^

According to the multivariate analysis, the risk of death was higher in patients with pre-existing heart disease, a low score for physical functioning, or symptoms of dysphagia and/or chest pain, as well as those who did not undergo surgical treatment. We recognize several reasons that justify our findings. First, it should be noted that cardiopathy is a frequent comorbidity in elderly patients with lung cancer and one of the major causes of death in the general population. Second, physical functioning is evaluating the patient fitness, considering that a better performance status is generally associated with better survival. Finally, chest pain and dysphagia are symptoms probably related to locoregionally advanced disease in this population and therefore associated with worse prognosis.

Future research on the use of geriatric evaluation in elderly lung cancer patients should be powered to understand how it could potentially contribute to optimal decision making.^32,43^ On the other hand, it can be difficult to conduct research on elderly patients, given the slow recruitment and strict selection criteria for inclusion of patients in trials.^44^ In our study, we prospectively assessed an unselected elderly population, an approach which may provide useful insights for daily routine clinical practice.

## Conclusions

Patients over 75 years of age tend to receive more conservative treatments, involving less surgery and less concomitant radiochemotherapy. The surgical modality improved survival and therefore, this treatment modality should be carefully considered on case-by-case basis, regardless of patient age. A history of comorbidities and poor baseline QOL according to the EORTC QLQc30 and LC-13 (low physical functioning, marked dysphagia, and chest pain) are predictive of shorter survival. Therefore, measuring these parameters before treatment may help us to define a population of frail patients with a poorer prognosis to facilitate decision making in clinical practice. Prospective studies in this crucial and understudied area are needed.

## References

[1] Available from: http://seer.cancer.gov/statfacts/html/lungb.html n.d

[2] Gridelli C, Langer C, Maione P, Rossi A, Schild SE . Lung cancer in the elderly. J Clin Oncol 2007; 25: 1898–1907. doi: 10.1200/JCO.2006.10.3085 17488989

[3] Brown S, Banfill K, Aznar MC, Whitehurst P, Faivre Finn C . The evolving role of radiotherapy in non-small cell lung cancer. Br J Radiol 2019; 92(1104): 20190524. doi: 10.1259/bjr.20190524 31535580 PMC6913359

[4] Shrimali RK, Chakraborty S, Prasath S, Arun B, Chatterjee S . Impact of modern radiotherapy techniques on survival outcomes for unselected patients with large volume non-small cell lung cancer. Br J Radiol 2019; 92(1095): 20180928. doi: 10.1259/bjr.20180928 30457882 PMC6540869

[5] Aupérin A, Le Péchoux C, Rolland E, Curran WJ, Furuse K, Fournel P, et al . Meta-analysis of concomitant versus sequential radiochemotherapy in locally advanced non-small-cell lung cancer. J Clin Oncol 2010; 28: 2181–90. doi: 10.1200/JCO.2009.26.2543 20351327

[6] Blanco R, Maestu I, de la Torre MG, Cassinello A, Nuñez I . A review of the management of elderly patients with non-small-cell lung cancer. Ann Oncol 2015; 26: 451–63. doi: 10.1093/annonc/mdu268 25060421

[7] Atagi S, Kawahara M, Yokoyama A, Okamoto H, Yamamoto N, Ohe Y, et al . Thoracic radiotherapy with or without daily low-dose carboplatin in elderly patients with non-small-cell lung cancer: a randomised, controlled, phase 3 trial by the japan clinical oncology group (JCOG0301). Lancet Oncol 2012; 13: 671–78. doi: 10.1016/S1470-2045(12)70139-0 22622008

[8] Joo JH, Song SY, Kim SS, Jeong Y, Jeong S-Y, Choi W, et al . Definitive radiotherapy alone over 60 gy for patients unfit for combined treatment to stage II-III non-small cell lung cancer: retrospective analysis. Radiat Oncol 2015; 10: 250. doi: 10.1186/s13014-015-0560-z 26635014 PMC4668693

[9] Sigel K, Lurslurchachai L, Bonomi M, Mhango G, Bergamo C, Kale M, et al . Effectiveness of radiation therapy alone for elderly patients with unresected stage III non-small cell lung cancer. Lung Cancer 2013; 82: 266–70. doi: 10.1016/j.lungcan.2013.06.011 24011407 PMC3845375

[10] de Rijke JM, Schouten LJ, ten Velde GPM, Wanders SL, Bollen ECM, Lalisang RI, et al . Influence of age, comorbidity and performance status on the choice of treatment for patients with non-small cell lung cancer; results of a population-based study. Lung Cancer 2004; 46: 233–45. doi: 10.1016/j.lungcan.2004.03.011 15474672

[11] Little AG, Gay EG, Gaspar LE, Stewart AK . National survey of non-small cell lung cancer in the united states: epidemiology, pathology and patterns of care. Lung Cancer 2007; 57: 253–60. doi: 10.1016/j.lungcan.2007.03.012 17451842

[12] Semrau S, Zettl H, Hildebrandt G, Klautke G, Fietkau R . Older patients with inoperable non-small cell lung cancer: long-term survival after concurrent chemoradiotherapy. Strahlenther Onkol 2014; 190: 1125–32. doi: 10.1007/s00066-014-0710-5 25098688

[13] Hsu C-L, Chen J-H, Chen K-Y, Shih J-Y, Yang J-H, Yu C-J, et al . Advanced non-small cell lung cancer in the elderly: the impact of age and comorbidities on treatment modalities and patient prognosis. J Geriatr Oncol 2015; 6: 38–45. doi: 10.1016/j.jgo.2014.09.178 25245172

[14] Wanders R, Steevens J, Botterweck A, Dingemans A-MC, Reymen B, Baardwijk A van, et al . Treatment with curative intent of stage III non-small cell lung cancer patients of 75 years: a prospective population-based study. Eur J Cancer 2011; 47: 2691–97. doi: 10.1016/j.ejca.2011.06.023 21733675

[15] Mellemgaard A, Lüchtenborg M, Iachina M, Jakobsen E, Green A, Krasnik M, et al . Role of comorbidity on survival after radiotherapy and chemotherapy for nonsurgically treated lung cancer. J Thorac Oncol 2015; 10: 272–79. doi: 10.1097/JTO.0000000000000416 25371078

[16] Antonio M, Saldaña J, Linares J, Ruffinelli JC, Palmero R, Navarro A, et al . Geriatric assessment may help decision-making in elderly patients with inoperable, locally advanced non-small-cell lung cancer. Br J Cancer 2018; 118: 639–47. doi: 10.1038/bjc.2017.455 29381689 PMC5846066

[17] American Joint Committee on Cancer. In: TNM lung cancer staging. 7th ed. Memorial Sloan-Kettering Cancer Center; 2009.

[18] Tammemagi CM, Neslund-Dudas C, Simoff M, Kvale P . Smoking and lung cancer survival: the role of comorbidity and treatment. Chest 2004; 125: 27–37. doi: 10.1378/chest.125.1.27 14718417

[19] Park SY, Lee JG, Kim J, Bae MK, Lee CY, Kim DJ, et al . The influence of smoking intensity on the clinicopathologic features and survival of patients with surgically treated non-small cell lung cancer. Lung Cancer 2013; 81: 480–86. doi: 10.1016/j.lungcan.2013.07.002 23896023

[20] Huang Y, Wei S, Jiang N, Zhang L, Wang S, Cao X, et al . The prognostic impact of decreased pretreatment haemoglobin level on the survival of patients with lung cancer: a systematic review and meta-analysis. BMC Cancer 2018; 18(1): 1235. doi: 10.1186/s12885-018-5136-5 30526532 PMC6288911

[21] Pauwels RA, Buist AS, Calverley PM, Jenkins CR, Hurd SS, GOLD Scientific Committee . Global strategy for the diagnosis, management, and prevention of chronic obstructive pulmonary disease. NHLBI/WHO global initiative for chronic obstructive lung disease (GOLD) workshop summary. Am J Respir Crit Care Med 2001; 163: 1256–76. doi: 10.1164/ajrccm.163.5.2101039 11316667

[22] Maione P, Perrone F, Gallo C, Manzione L, Piantedosi F, Barbera S, et al . Pretreatment quality of life and functional status assessment significantly predict survival of elderly patients with advanced non-small-cell lung cancer receiving chemotherapy: a prognostic analysis of the multicenter italian lung cancer in the elderly study. J Clin Oncol 2005; 23: 6865–72. doi: 10.1200/JCO.2005.02.527 16192578

[23] Efficace F, Bottomley A, Smit EF, Lianes P, Legrand C, Debruyne C, et al . Is A patient’s self-reported health-related quality of life A prognostic factor for survival in non-small-cell lung cancer patients? A multivariate analysis of prognostic factors of EORTC study 08975. Ann Oncol 2006; 17: 1698–1704. doi: 10.1093/annonc/mdl183 16968876

[24] Quinten C, Martinelli F, Coens C, Sprangers MAG, Ringash J, Gotay C, et al . A global analysis of multitrial data investigating quality of life and symptoms as prognostic factors for survival in different tumor sites. Cancer 2014; 120: 302–11. doi: 10.1002/cncr.28382 24127333

[25] Fayers P, Bottomley A . European Organisation for Research and Treatment of Cancer Eur J Cancer 2002; ;38 Suppl 4:. Available from: 10.1016/s0959-8049(01)00448-8 11858978

[26] Bergman B, Aaronson NK, Ahmedzai S, Kaasa S, Sullivan M . The EORTC QLQ-LC13: a modular supplement to the EORTC core quality of life questionnaire (QLQ-C30) for use in lung cancer clinical trials. EORTC study group on quality of life. Eur J Cancer 1994; 30A: 635–42. doi: 10.1016/0959-8049(94)90535-5 8080679

[27] Ediebah DE, Quinten C, Coens C, Ringash J, Dancey J, Zikos E, et al . Quality of life as A prognostic indicator of survival: A pooled analysis of individual patient data from canadian cancer trials group clinical trials. Cancer 2018; 124: 3409–16. doi: 10.1002/cncr.31556 29905936

[28] Spoelstra FOB, Senan S, Le Péchoux C, Ishikura S, Casas F, Ball D, et al . Variations in target volume definition for postoperative radiotherapy in stage III non-small-cell lung cancer: analysis of an international contouring study. Int J Radiat Oncol Biol Phys 2010; 76: 1106–13. doi: 10.1016/j.ijrobp.2009.02.072 19560881

[29] Steenbakkers R, Duppen J, Fitton I, Deurloo K, Nowak P, van Herk M, et al . A 3D analysis and reduction of observer variation in delineation of lung cancer for radiotherapy. International Journal of Radiation Oncology*Biology*Physics 2004; 60: S531–32. doi: 10.1016/j.ijrobp.2004.07.490

[30] Segawa K . Scanning electron microscopic studies on the iridocorneal angle tissue in normal human eyes. Nippon Ganka Gakkai Zasshi 1972; 76: 659–63. 4674264

[31] Bentzen SM, Constine LS, Deasy JO, Eisbruch A, Jackson A, Marks LB, et al . Quantitative analyses of normal tissue effects in the clinic (QUANTEC): an introduction to the scientific issues. Int J Radiat Oncol Biol Phys 2010; 76: S3-9. doi: 10.1016/j.ijrobp.2009.09.040 20171515 PMC3431964

[32] Driessen EJM, Bootsma GP, Hendriks LEL, van den Berkmortel F, Bogaarts B, van Loon JGM, et al . Stage III non-small cell lung cancer in the elderly: patient characteristics predictive for tolerance and survival of chemoradiation in daily clinical practice. Radiother Oncol 2016; 121: 26–31. doi: 10.1016/j.radonc.2016.07.025 27522577

[33] Driessen EJM, Schulkes KJG, Dingemans A-M, van Loon JGM, Hamaker ME, Aarts MJ, et al . Patterns of treatment and survival among older patients with stage III non-small cell lung cancer. Lung Cancer 2018; 116: 55–61. doi: 10.1016/j.lungcan.2017.12.013 29413051

[34] Vyfhuis MAL, Bentzen SM, Molitoris JK, Diwanji T, Badiyan S, Grover S, et al . Patterns of care and survival in stage III NSCLC among black and latino patients compared with white patients. Clin Lung Cancer 2019; 20: 248–57. doi: 10.1016/j.cllc.2019.02.015 30910573

[35] De Ruysscher D, Botterweck A, Dirx M, Pijls-Johannesma M, Wanders R, Hochstenbag M, et al . Eligibility for concurrent chemotherapy and radiotherapy of locally advanced lung cancer patients: a prospective, population-based study. Ann Oncol 2009; 20: 98–102. doi: 10.1093/annonc/mdn559 18718891

[36] Dawe DE, Christiansen D, Swaminath A, Ellis PM, Rothney J, Rabbani R, et al . Chemoradiotherapy versus radiotherapy alone in elderly patients with stage III non-small cell lung cancer: A systematic review and meta-analysis. Lung Cancer 2016; 99: 180–85. doi: 10.1016/j.lungcan.2016.07.016 27565937

[37] Movsas B, Moughan J, Sarna L, Langer C, Werner-Wasik M, Nicolaou N, et al . Quality of life supersedes the classic prognosticators for long-term survival in locally advanced non-small-cell lung cancer: an analysis of RTOG 9801. J Clin Oncol 2009; 27: 5816–22. doi: 10.1200/JCO.2009.23.7420 19858383 PMC2793002

[38] Qi Y, Schild SE, Mandrekar SJ, Tan AD, Krook JE, Rowland KM, et al . Pretreatment quality of life is an independent prognostic factor for overall survival in patients with advanced stage non-small cell lung cancer. J Thorac Oncol 2009; 4: 1075–82. doi: 10.1097/JTO.0b013e3181ae27f5 19546817 PMC2954489

[39] Grose D, Morrison DS, Devereux G, Jones R, Sharma D, Selby C, et al . The impact of comorbidity upon determinants of outcome in patients with lung cancer. Lung Cancer 2015; 87: 186–92. doi: 10.1016/j.lungcan.2014.11.012 25498829

[40] Firat S, Byhardt RW, Gore E . The effects of comorbidity and age on RTOG study enrollment in stage III non-small cell lung cancer patients who are eligible for RTOG studies. Int J Radiat Oncol Biol Phys 2010; 78: 1394–99. doi: 10.1016/j.ijrobp.2009.09.051 20646856

[41] Kale MS, Mhango G, Gomez JE, Sigel K, Smith CB, Bonomi M, et al . Treatment toxicity in elderly patients with advanced non-small cell lung cancer. Am J Clin Oncol 2017; 40: 470–76. doi: 10.1097/COC.0000000000000188 25784564 PMC4568179

[42] Coate LE, Massey C, Hope A, Sacher A, Barrett K, Pierre A, et al . Treatment of the elderly when cure is the goal: the influence of age on treatment selection and efficacy for stage III non-small cell lung cancer. J Thorac Oncol 2011; 6: 537–44. doi: 10.1097/JTO.0b013e31820b8b9b 21258243

[43] Hamaker ME, Te Molder M, Thielen N, van Munster BC, Schiphorst AH, van Huis LH . The effect of A geriatric evaluation on treatment decisions and outcome for older cancer patients - A systematic review. J Geriatr Oncol 2018; 9: 430–40. doi: 10.1016/j.jgo.2018.03.014 29631898

[44] Schulkes KJG, Nguyen C, van den Bos F, van Elden LJR, Hamaker ME . Selection of patients in ongoing clinical trials on lung cancer. Lung 2016; 194: 967–74. doi: 10.1007/s00408-016-9943-7 27650509

